# MicroRNA-3127 promotes cell proliferation and tumorigenicity in hepatocellular carcinoma by disrupting of PI3K/AKT negative regulation

**DOI:** 10.18632/oncotarget.3438

**Published:** 2015-01-31

**Authors:** Jianxin Jiang, Yi Zhang, Yuting Guo, Chao Yu, Meiyuan Chen, Zhu Li, Se Tian, Chengyi Sun

**Affiliations:** ^1^ Department of Hepatobiliary Surgery, Affiliated Hospital of Guiyang Medical College, Guiyang, China; ^2^ Zhongnan Hospital of Wuhan University, Institute of Hepatobiliary Diseases of Wuhan University, Transplant Center of Wuhan University, Hubei Key Laboratory of Medical Technology on Transplantation, Wuhan, China

**Keywords:** Hepatocellular carcinoma, miR-3127, PI3K/AKT pathway, PHLPP, INPP4A

## Abstract

Recent studies have shown that multiple phosphatases deactivate the PI3K/AKT signaling pathway. Here we demonstrated that, by suppressing multiple phosphatases, miR-3127 promotes growth of hepatocellular carcinoma (HCC). Our study also reveals clinical significance of miR-3127 expression in HCC patients. MiR-3127 expression was markedly upregulated in HCC tissues and cells. Furthermore, high miR-3127 expression was associated with an aggressive phenotype and poor prognosis. MiR-3127 overexpression promoted HCC cell proliferation *in vitro* and tumor growth *in vivo*. Also, miR-3127 accelerated G1-S transition by activating AKT/FOXO1 signaling, by directly targeting the 3′ untranslated regions (3`UTR) of pleckstrin homology domain leucine-rich repeat protein phosphatase 1/2 (PHLPP1/2), inositol polyphosphate phosphatase 4A (INPP4A), and inositol polyphosphate-5-phosphatase J (INPP5J) mRNA, repressing their expression. In agreement, the miRNA antagonist antagomir-3127 suppressed HCC cell proliferation and tumor growth by inhibiting the AKT/FOXO1 signaling. Taken together, these findings suggest that silencing miR-3127 might be a potential therapeutic strategy.

## INTRODUCTION

Hepatocellular carcinoma (HCC) is the main type of liver cancer and is the sixth most common malignancy and the third leading cause of cancer death worldwide [1]. Despite advances in HCC diagnosis and treatment in recent decades, there have been few significant improvements in overall survival: the 5-year survival rate remains below 30% in HCC patients after surgical resection, mainly due to the high rate of recurrence [2, 3]. Thus, understanding the mechanisms underlying HCC is essential for developing novel therapeutic strategies.

Recent advances have shown that phosphatidylinositol 3-kinase/v-akt murine thymoma viral oncogene homolog (PI3K/AKT) pathway alterations play an important role in the development of a variety of human carcinomas, including HCC [4-6]. The PI3K/AKT pathway is a key signal transduction system that links oncogenes and multiple receptor classes to many essential cellular functions, and PI3K/AKT pathway activation promotes HCC cell tumorigenicity [7-9]. Conversely, inhibiting PI3K/AKT signaling markedly suppresses proliferation and is considered a therapeutic approach in HCC [10]. Generally, the AKT cascade is activated by receptor tyrosine kinases, cytokine receptors, G protein–coupled receptors, and other stimuli that induce the production of phosphatidylinositol-3,4,5-triphosphates [PI(3,4,5)P3] by PI3K [11]. The activated AKT further phosphorylates multiple downstream effectors, such as glycogen synthase kinase-3 (GSK3α/β) and forkhead transcription factor (FoxO) family members, which together transmit potent growth, proliferation, and survival signals to cells [12-14].

Under physiological conditions, the strength and duration of activated PI3K/AKT signaling is tightly regulated by multiple phosphoinositide and protein phosphatases, including phosphatase and tensin homologue (PTEN) [15], pleckstrin homology domain leucine-rich repeat protein phosphatase (PHLPP) [16], inositol polyphosphate phosphatase 4A (INPP4A) [17, 18], and inositol polyphosphate-5-phosphatase J (INPP5J) [19]. Phosphoinositide phosphatases negatively regulate PI(3,4,5)P3 levels by removing the phosphate of PI(3,4,5)P3, and protein phosphatases dephosphorylate and inactivate AKT, ultimately leading to the termination of the PI3K/AKT signaling. However, emerging evidence shows that the PI3K/AKT signaling pathway is constitutively activated in a wide range of tumor types [20]. Aside from these mechanisms, the functional loss of phosphatases can lead to constitutively activated PI3K/AKT signaling. The tumor suppressor PTEN is frequently deleted and mutated in human tumors [21], and PTEN loss of function led to AKT hyperactivation and increased tumorigenesis in mice [22]. However, the loss-of-function mechanism of other phosphatases, including PHLPP, INPP4A, and INPP5J, has not been fully elucidated. How cancer cells simultaneously override these negative regulations to constitutively activate PI3K/AKT require further investigation.

Herein, we found that microRNA-3127 (miR-3127) was overexpressed in HCC and correlated with poor patient prognosis. MiR-3127 upregulation sustained PI3K/AKT signaling by directly suppressing PHLPP1, PHLPP2, INPP4A, and INPP5J expression, and induced an aggressive phenotype of HCC both *in vitro* and *in vivo*. We uncovered a novel mechanism that dysregulates the precise balance between PI3K/AKT phosphorylation and dephosphorylation in HCC, supporting the clinical and functional significance of epigenetic events in cancer progression.

## RESULTS

### MiR-3127 is upregulated in HCC tissues and cell lines and associated with poor prognosis

**Figure 1:**
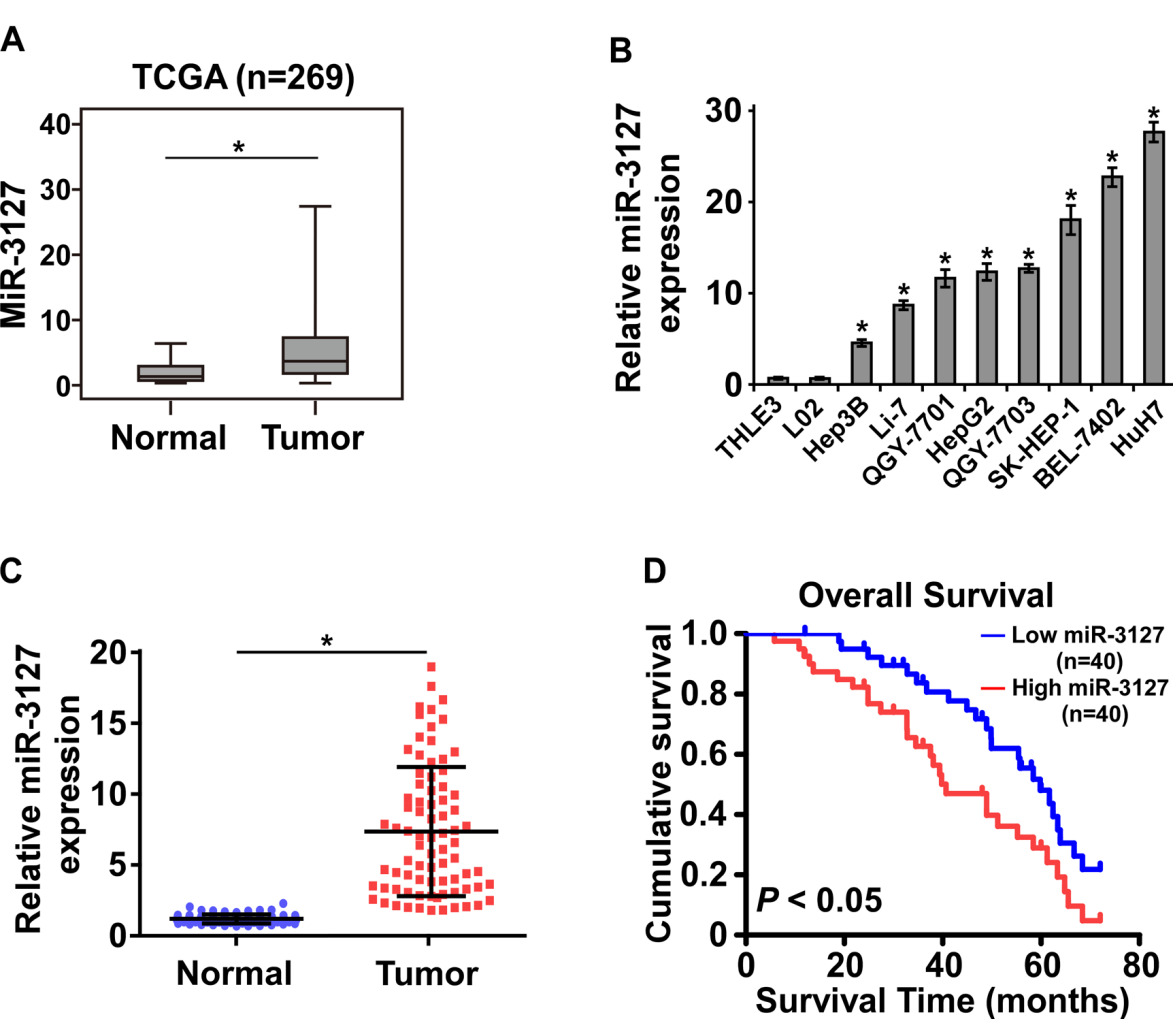
MiR-3127 was upregulated in HCC tissues and cell lines and associated with poor prognosis A. Upregulated miR-3127 in primary HCC tissues (Tumor) compared with normal esophageal tissue (Normal) (n = 269, TCGA). B. MiR-3127 expression levels in HCC cell lines (BEL-7402, Hep3B, HepG2, Huh7, Li-7, QGY-7701, QGY-7703, SK-HEP-1) and normal liver epithelial cells (THLE-3, LO2). U6 was used as the RNA loading control; miRNA levels were normalized to that of U6 RNA. Bars represent the mean ± SD of three independent experiments. C. Real-time PCR analysis of miR-3127 expression in primary HCC tissues (Tumor) with matched adjacent non-tumor tissues (Normal) from 80 individual patients. D. Kaplan–Meier analysis of overall survival stratified by low miR-3127 expression (<median, n = 40, blue) and high miR-3127 expression (>median, n = 40, red). MiR-3127 upregulation was significantly correlated with shorter overall survival. *P < 0.05.

By analyzing the miRNA sequencing datasets of 269 HCC cases downloaded from The Cancer Genome Atlas (TCGA), we found that miR-3127 expression was upregulated in primary HCC tissues compared with normal liver tissue (Fig. 1A). To validate the miR-3127 upregulation detected in HCC, real-time PCR was performed on HCC cell lines and clinical samples. As shown in (Fig. 1B-C), miR-3127 expression was differentially upregulated in HCC cell lines and the primary HCC tissues from 80 individual patients compared with that in two normal liver cell lines and matched adjacent normal tissues, respectively. Therefore, the published miRNA datasets and our results suggest that miR-3127 is upregulated in HCC.

To investigate whether upregulated miR-3127 is involved in HCC progression, we examined the correlation between miR-3127 levels and the clinicopathological features of 80 patients with HCC. Statistical analyses showed that miR-3127 upregulation was significantly associated with larger tumor size (*P* = 0.012), poorer histological grade (*P* = 0.041), and higher clinical stage (*P* = 0.030) (Supplementary Table 3). Importantly, Kaplan–Meier survival analysis revealed that patients with upregulated miR-3127 had shorter overall survival (Fig. 1D). The median survival time of patients whose tumors showed high expression levels of miR-3127 was only 40.7 months, whereas the median survival time of those with low levels of miR-1181 expression was 59.8 months. These results show that upregulated miR-3127 is associated with poor prognosis.

### MiR-3127 upregulation promotes HCC cell proliferation *in vitro*

**Figure 2:**
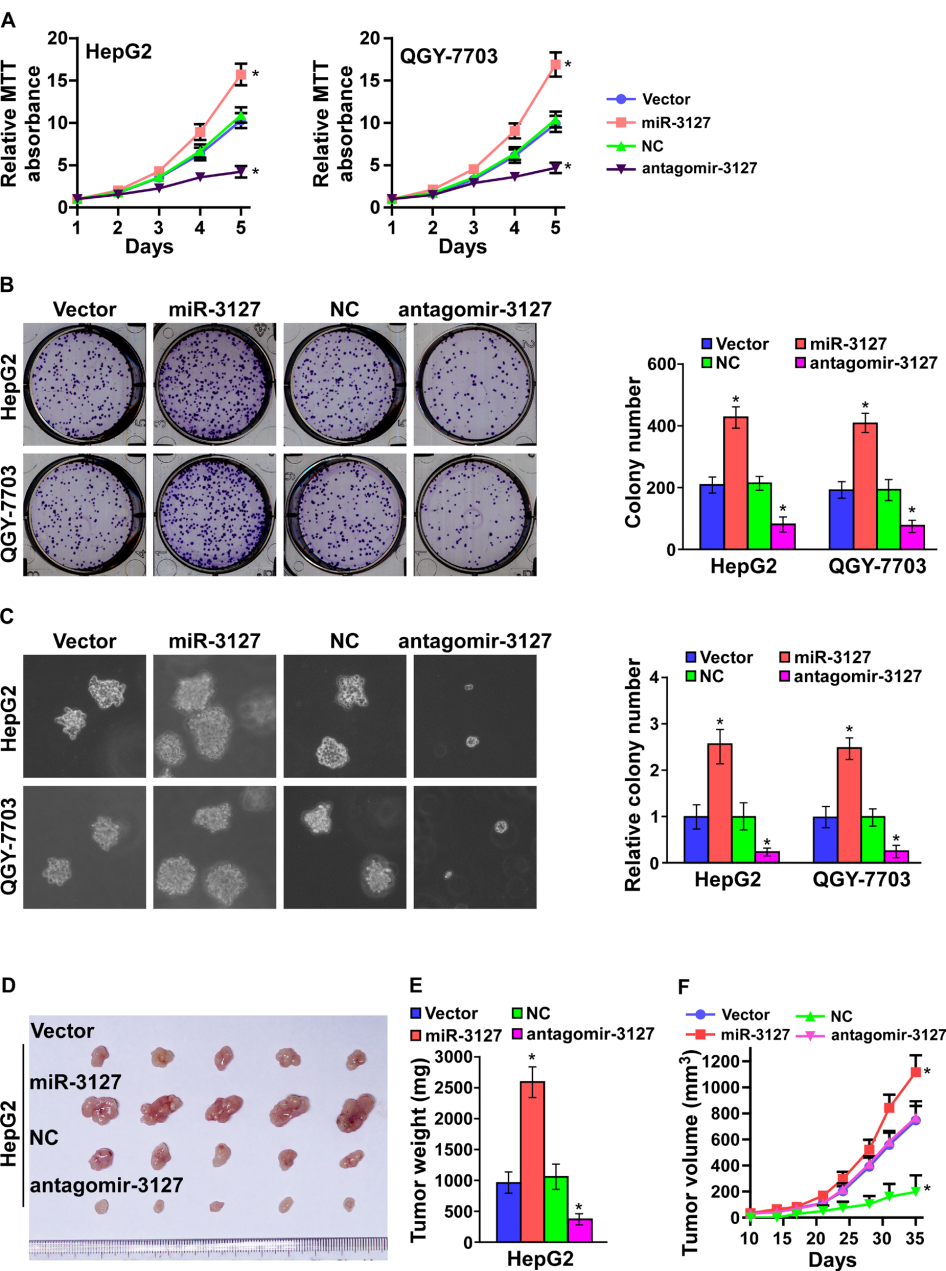
MiR-3127 upregulation promoted HCC cell proliferation *in vitro* and tumorigenicity*in vivo* A. MTT assay revealing that miR-3127 upregulation promoted HepG2 and QGY-7703 cell growth. B. Representative micrographs (left) and quantification (right) of crystal violet–stained cell colonies. C. Representative micrographs (left) at 100-fold magnification and quantification (right) of colonies in the anchorage-independent growth assay. Colonies > 0.1 mm in diameter were scored. D. Tumors from mice in each group (n = 5 per group). E. Growth curves for tumor formation after implantation of HepG2 cells. Mean tumor volumes are plotted. F. Histograms of the mean tumor weights of each group. Bars represent the mean ± SD of three independent experiments. *P < 0.05. NC, negative control.

As miR-3127 upregulation was correlated with clinical stage and tumor size in HCC clinical tissues, we examined whether miR-3127 is involved in HCC proliferation. HepG2 and QGY-7703 cells were engineered to stably overexpress miR-3127, and we used the miRNA antagonist antagomir-3127 to silence endogenous miR-3127 (Supplementary Fig. 1 and Supplementary Fig. 2A). MTT and colony formation assays revealed that miR-3127 upregulation promoted HepG2 and QGY-7703 cell growth *in vitro* (Fig. 2A-B). Furthermore, we found that the anchorage-independent growth activity of HepG2 and QGY-7703 cell was dramatically enhanced by miR-3127 overexpression, as indicated by the increased colony numbers on soft agar (Fig. 2C). Importantly, downregulated miR-3127 drastically inhibited HCC cell proliferation *in vitro* (Fig. 2A-C). These results show that miR-3127 overexpression promotes HCC cell proliferation *in vitro* and that silencing miR-3127 inhibits HCC cell proliferation ability.

### Silenced endogenous miR-3127 inhibits HCC cell tumorigenicity *in vivo*

To test whether miR-3127 could enhance HCC cell tumorigenesis, miR-3127–overexpressing and vector control cells were inoculated into nude mice. As shown in (Fig. 2D-F), the tumors formed by miR-3127–overexpressing HepG2 cells were larger and heavier than the control tumors. We also investigated whether antagomir-3127 could inhibit the tumorigenicity of HCC cells. Nude mice bearing HepG2-derived tumors were injected with antagomir-3127 or negative control through the lateral tail vein every three days, and the tumors were smaller and lighter than the control tumors after 4 weeks (Fig. 2D-F). Taken together, these results indicate that miR-3127 overexpression promotes HCC cell proliferation *in vivo* and that silencing miR-3127 inhibits HCC cell tumorigenic ability.

### MiR-3127 downregulation inhibits cell cycle progression of HCC cells

**Figure 3:**
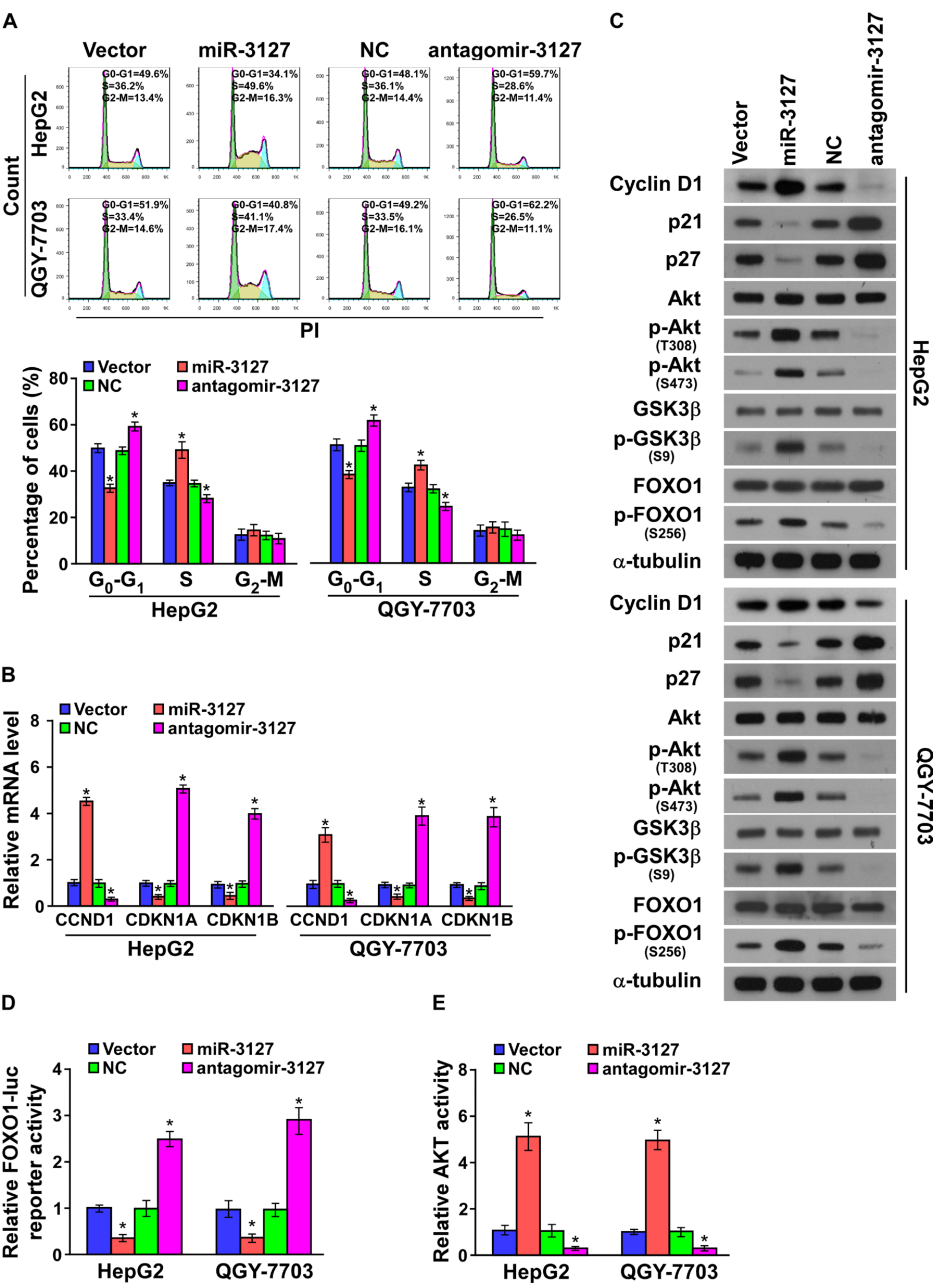
MiR-3127 downregulation inhibited cell cycle progression of HCC cells A. Flow cytometric analysis of HepG2 and QGY-7703 cells. B. Real-time PCR analysis of *CCND1*, *CDKN1A*, and *CDKN1B* mRNA expression in HepG2 and QGY-7703 cells. *GAPDH* served as the loading control. C. Western blotting analysis of cyclin D1, p21, p27, AKT, phosphorylated (p)-AKT (T308), FOXO1, p-FOXO1 (S256), GSK3β, and p-GSK3β (S9) protein in HepG2 and QGY-7703 cells. α-Tubulin served as the loading control. D. Analysis of HepG2 and QGY-7703 cell FOXO1 activity. E. Analysis of HepG2 and QGY-7703 cell AKT activity. Bars represent the mean ± SD of three independent experiments. *P < 0.05. NC, negative control.

We further investigated the mechanism underlying the miR-3127 silencing-mediated inhibition of HCC cell proliferation. As shown in (Fig. 3A), flow cytometry showed that miR-3127 downregulation dramatically decreased the percentage of cells in the S phase and increased that of cells in the G_1_/G_0_ phase, whereas upregulated miR-3127 increased the percentage of cells in the S phase and decreased that of cells in the G_1_/G_0_ phase, suggesting that antagomir-3127 might result in G_1_/S arrest in HCC cells. Furthermore, the expression levels of a number of critical cell cycle regulators were detected. As shown in (Fig. 3B-C), silencing miR-3127 resulted in downregulation of cyclin D1 (CCND1), whereas p21 (cyclin-dependent kinase inhibitor 1A, CDKN1A) and p27 (CDKN1B) were strikingly downregulated at both protein and mRNA level. MiR-3127 overexpression upregulated cyclin D1 expression, while p21 and p27 protein and mRNA were increased (Fig. 3B-C).

It has been well documented that CDKN1A [23], CDKN1B [24], and CCND1 [25] expression can be transcriptionally regulated by forkhead box O1 (FOXO1), and the transcriptional activity of FOXO1 is in turn modulated by AKT phosphorylation [26, 27]. Thus, we hypothesized that miR-3127 upregulation may activate PI3K/AKT/FOXO1 signaling. As shown in (Fig. 3C), the levels of p-FOXO1 (S256), p-AKT (T308), p-AKT (S473), and p-GSK3β (S9) were drastically increased in miR-3127–overexpressing HCC cells, while silencing miR-3127 decreased them (Fig. 3C). Moreover, FOXO1 activity was strongly repressed by miR-3127 overexpression, whereas miR-3127 silencing increased FOXO1 transcriptional regulatory activity (Fig. 3D). Consistently, AKT activity was significantly induced in miR-3127–overexpressing cells but was decreased in antagomir-3127–transfected cells (Fig. 3E). These results suggest that silencing miR-3127 inhibits the cell cycle progression of HCC cells and blocks PI3K/AKT/FOXO1 signaling.

### MiR-3127 activates PI3K/AKT pathway by targeting multiple negative regulators

**Figure 4:**
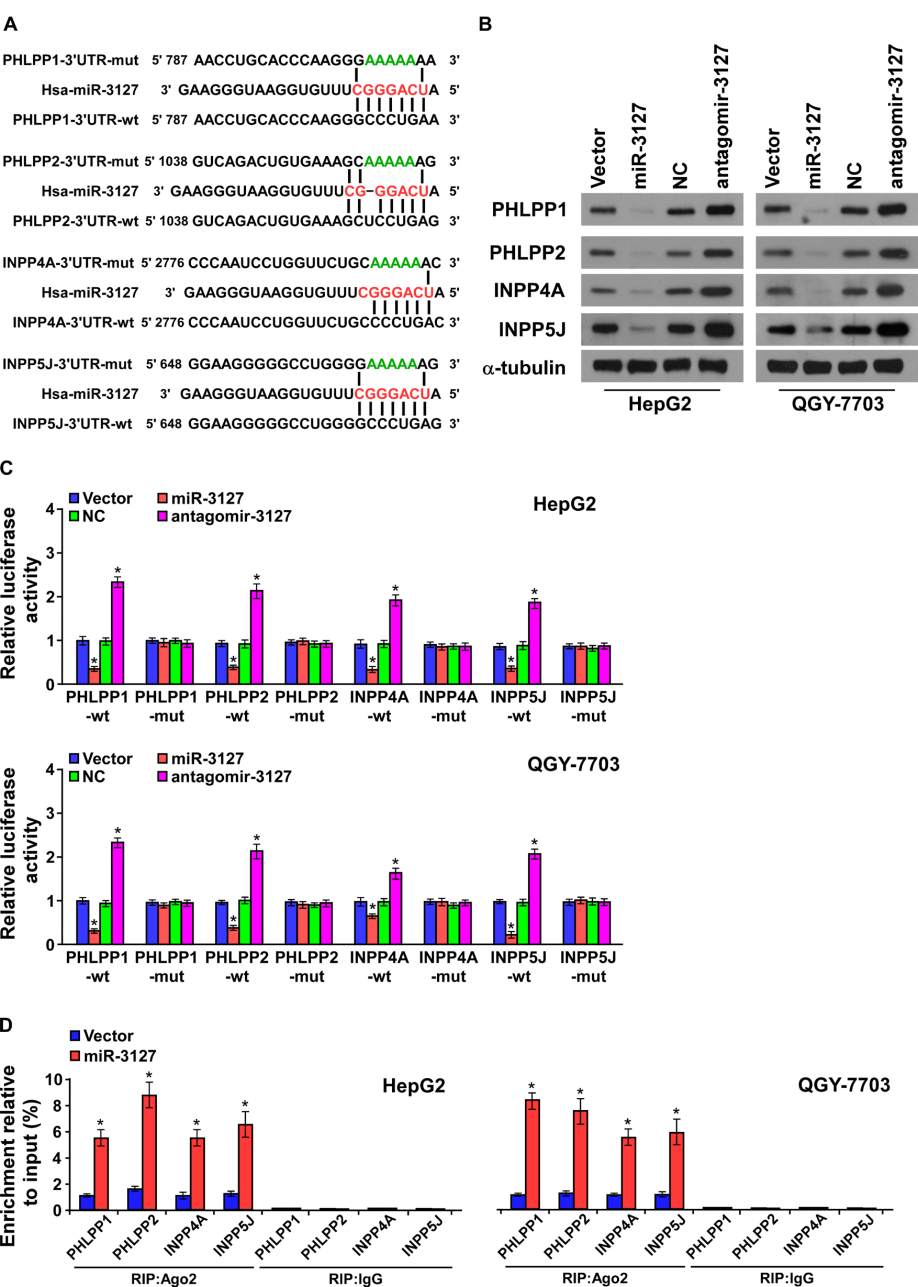
MiR-3127 promoted the PI3K/AKT signaling pathway by targeting *PHLPP1*, *PHLPP2*, *INPP4A*, and *INPP5J* A. The predicted miR-3127 target sequence in the wild-type (wt) 3′ UTR of *PHLPP1*, *PHLPP2*, *INPP4A*, and *INPP5J*. Mutation (mut) of the four mRNA 3′ UTR sequences had similar sequences containing five point mutations (green) in the miR-3127 seed sequence. B. Western blotting of PHLPP1, PHLPP2, INPP4A, and INPP5J expression in HepG2 and QGY-7703 cells. α-tubulin was used as the loading control. C. Luciferase assay of HepG2 and QGY-7703 cells transfected with pGL3-3UTR reporter and miR-3127 or antagomir-3127. D. RIP analysis, as assessed by Ago2 immunoprecipitation in HepG2 and QGY-7703 cells. Immunoglobulin G (IgG) immunoprecipitation was used as the negative control. Bars represent the mean ± SD of three independent experiments. *P < 0.05. NC, negative control.

Analysis using publically available algorithms showed that *PHLPP1*, *PHLPP2*, *INPP4A*, and *INPP5J* might be potential targets of miR-3127 (miRanda, TargetScan; Fig. 4A). As predicted, western blotting revealed that PHLPP1, PHLPP2, INPP4A, and INPP5J expression was decreased in miR-3127–upregulated HepG2 and QGY-7703 cells but was increased following antagomir-3127 transfection *in vitro* (Fig. 4B) and *in vivo* (Supplementary Fig. 2B). Luciferase reporter analysis showed that miR-3127 overexpression reduced the luciferase reporter activity of the *PHLPP1*, *PHLPP2*, *INPP4A*, and *INPP5J* 3′ UTR, but that antagomir-3127 increased it. However, the luciferase reporter activity of the 3′ UTR of the four genes that contained point mutations (mut) in the miR-3127–binding seed region was unaffected by miR-3127 overexpression or antagomir-3127 treatment (Fig. 4C). To confirm that miR-3127 directly interacts with *PHLPP1*, *PHLPP2*, *INPP4A*, and *INPP5J* mRNA, we tested whether miR-3127 mediated the RNA-induced silencing complex (RISC) binding to these four mRNA using miRNP immunoprecipitation assay. As shown in (Fig. 4D), miR-3127 overexpression increased *PHLPP1*, *PHLPP2*, *INPP4A*, and *INPP5J* mRNA binding with the RISC. Taken together, these results suggest that miR-3127 inhibits the PI3K/AKT signaling pathway by targeting *PHLPP1*, *PHLPP2*, *INPP4A*, and *INPP5J*.

### Blocking PI3K/AKT signaling suppresses miR-3127–induced proliferation

**Figure 5:**
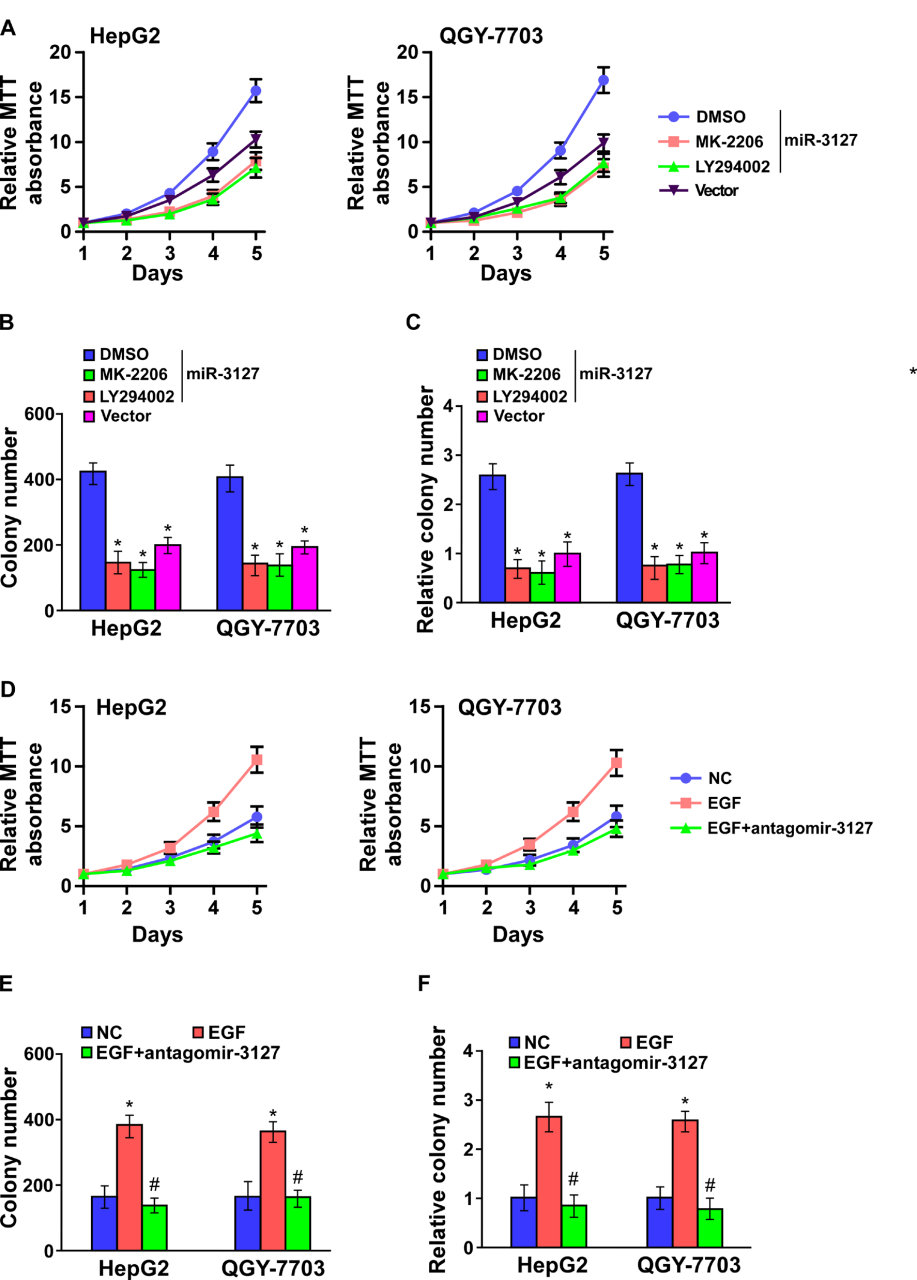
Blocked PI3K/AKT signaling suppressed miR-3127–induced proliferation A–C. AKT inhibitor (MK-2206, 1 μM) and PI3K inhibitor (LY294002, 10 μM) suppressed the miR-3127–induced proliferation in HCC cells as determined by (A) MTT assay, (B) colony formation assay, and (C) anchorage-independent growth assay. Scale bars, 100 mm. Colonies > 0.1 mm in diameter were scored. D–F. Silencing miR-3127 inhibited HCC cell proliferation induced by EGF (10 ng/ml) as determined by (D) MTT assay, (E) colony formation assay, and (F) anchorage-independent growth assay in low serum culture condition (1% FBS). Bars represent the mean ± SD of three independent experiments. *P < 0.05. DMSO, dimethyl sulfoxide; NC, negative control.

To confirm that miR-3127 upregulation promotes HCC proliferation by activating PI3K/AKT signaling, miR-3127–transfected HepG2 and QGY-7703 cells were treated with an AKT inhibitor (MK-2206) and an PI3K inhibitor (LY294002). As shown in (Fig. 5A-C), HCC cell proliferation rate and colony formation and anchorage-independent growth abilities were dramatically decreased. Importantly, miR-3127 silencing resulted in epidermal growth factor (EGF)-induced downregulated HCC cell proliferation activity in low serum culture condition (Fig. 5D-F) suggesting that silencing miR-3127 inhibits HCC cell proliferation by blocking PI3K/AKT signaling.

### MiR-3127 levels correlate with PHLPP1, PHLPP2, INPP4A, and INPP5J expression in clinical HCC tissues

**Figure 6:**
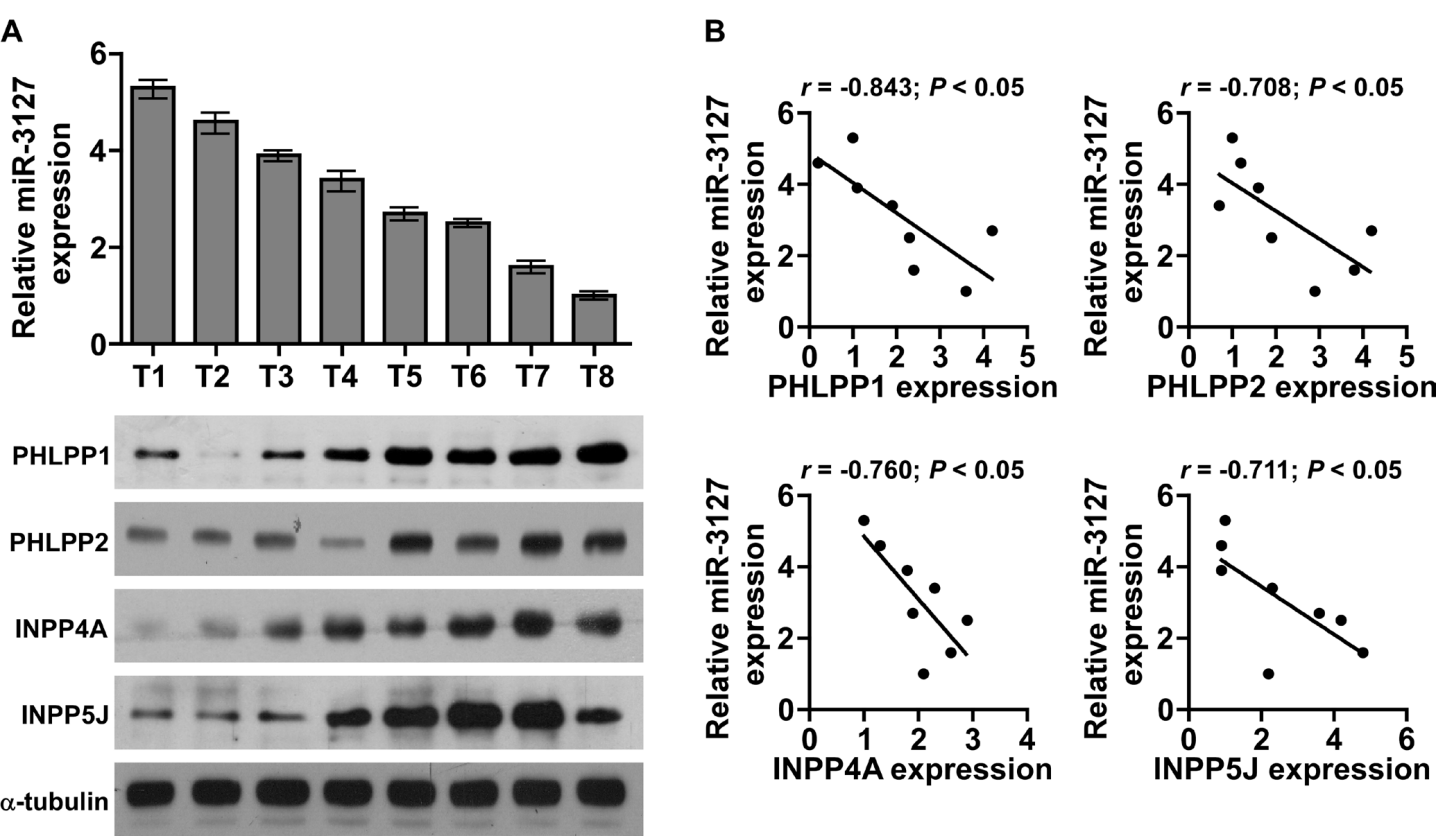
MiR-3127 levels were correlated with *PHLPP1*, *PHLPP2*, *INPP4A*, and *INPP5J* expression in HCC clinical tissues A. Real-time PCR of miR-3127 expression and western blotting of PHLPP1, PHLPP2, INPP4A, and INPP5J expression in eight fresh HCC tissue specimens (T). U6 was used as the RNA loading control; miRNA levels were normalized to that of miR-3127 expression in sample T8. Bars represent the mean ± SD of three independent experiments. α-Tubulin was used as the protein loading control. B. Correlation between miR-3127 levels and expression of the *PHLPP1*, *PHLPP2*, *INPP4A*, and *INPP5J* genes in HCC clinical tissues. Protein levels were normalized to that of expression in sample T1.

Next, we further investigated whether miR-3127 correlated with PHLPP1, PHLPP2, INPP4A, and INPP5J expression in HCC clinical tissues in eight fresh HCC tissues. As shown in (Fig. 6A-6B), a significant inverse correlation was found between miR-3127 and PHLPP1 (*r* = −0.843; *P* < 0.05), PHLPP2 (*r* = −0.708; *P* < 0.05), INPP4A (*r* = −0.760; *P* < 0.05), and INPP5J (*r* = −0.711; *P* < 0.05) expression in HCC (Fig. 6C). Taken together, these results indicate that PHLPP1, PHLPP2, INPP4A, and INPP5J expression correlates with miR-3127 levels in HCC tissues.

## DISCUSSION

A central transducer of growth and proliferative signaling, the PI3K/AKT signaling pathway plays an essential role in maintaining tumor cell proliferation, and constitutive activation of PI3K/AKT signaling is involved in the initiation and progression of various human cancers, resulting in poor prognosis [28, 29]. However, as both lipid and protein phosphatases antagonize this pathway, how cancer cells concomitantly supersede the negative regulation of phosphatases at diverse levels remains puzzling. Our results revealed that miR-3127 is substantially overexpressed in HCC, sustaining PI3K/AKT signaling by directly suppressing multiple phosphatases, including PHLPP1, PHLPP2, INPP4A, and INPP5J. Therefore, our findings suggest a novel mechanism that dysregulates the precise balance between PI3K/AKT pathway phosphorylation and dephosphorylation in HCC, and this mechanism is critical for developing new therapeutic strategies for HCC.

An increasing number of studies has shown that lipid and protein phosphatase deregulation plays an important role in cancer development and progression through the constitutive activation of PI3K/AKT signaling. PHLPP, a family of Ser/Thr phosphatases, negatively regulate the PI3K oncogenic pathway [30]. There are two PHLPP isozymes: PHLPP1 and PHLPP2, and they share a common architecture, including a phosphatase domain that is 58% conserved between PHLPP1 and PHLPP2 [16]. Biochemical and cellular validation studies have determined that PHLPP1 and PHLPP2 are functional phosphatases that dephosphorylate and inactivate AKT at its hydrophobic motif site, Ser473 [31, 32]. Previous reports have mentioned that PHLPP overexpression in glioblastoma, breast, colon, and pancreatic cancer cells decreases growth *in vitro* and in xenograft models [33-35]. Furthermore, PHLPP is downregulated in many tumors, and correlates with the growth, tumorigenesis, and metastasis potential of cancer [36, 37], which suggests that PHLPP plays an important role as a negative regulator of the PI3K/AKT pathway in cancer. However, the molecular mechanism of PHLPP loss of function in HCC is not clear; our data suggest that miR-3127 upregulation might contribute to downregulating PHLPP1 and PHLPP2 expression in HCC.

In addition, our findings showed that miR-3127 could also suppress the two phosphoinositide phosphatases, INPP4A and INPP5J, by directly targeting their 3′ UTR. INPP4A negatively regulates the levels of PI(3,4)P2, an AKT activator like PI(3,4,5)P3, by removing the phosphate of PI(3,4)P2 to yield PI(3)P. INPP4A knockout sustained AKT activation and thereby promoted mouse embryonic fibroblast proliferation, survival, and tumorigenesis [18, 38]. It has been noted that INPP5J suppresses PI3K/AKT signaling by reducing AKT at its hydrophobic motif site, Ser473, and was recently found to be downregulated in melanoma and esophageal squamous cell carcinoma, leading to the promotion of cell proliferation and tumorigenicity *in vivo* [19, 39]. In addition, our results showed that silencing endogenous miR-3127 upregulated PHLPP1, PHLPP2, INPP4A, and INPP5J protein levels, and inhibited HCC cell proliferation by suppressing the PI3K/AKT signaling pathway. Therefore, our findings suggest that antagomir-3127 might be developed as a new therapeutic strategy by recovering the PI3K/AKT signaling pathway negative regulators in HCC.

As the central effect on cancer initiation and progression, the PI3K/AKT pathway inhibitors have been integrated into clinical practice. However, developing methods for identifying patients with tumors ‘driven’ by molecular abnormalities might further enhance the anti-cancer effect of these inhibitors [15, 40, 41]. Herein, our results showed that PI3K/AKT inhibitors suppressed the miR-3127–induced proliferation, and miR-3127 levels were correlated with the number of phosphatases expressed in HCC clinical tissues. Whether miR-3127 expression reflects PI3K/AKT pathway activity levels and whether it is an effective molecular marker for the clinical application of PI3K/AKT pathway inhibitors warrants further research.

In conclusion, miR-3127 overexpression suppresses multiple phosphoinositide phosphatases and activates PI3K/AKT signaling. Silencing miR-3127 dramatically inhibits HCC cell proliferation and tumorigenicity by inhibiting the cell cycle progression of HCC cells. Further exploration of the precise role of miR-3127 in the pathogenesis of a variety of tumors and in PI3K/AKT signaling pathway activation will increase our knowledge of the molecular regulation of cancer progression and may allow the development of new therapeutic strategies against HCC.

## MATERIALS AND METHODS

### Cell lines and cell culture

Human HCC cell lines (BEL-7402, Hep3B, HepG2, HuH7, Li-7, QGY-7701, QGY-7703, and SK-HEP-1) and immortalized normal liver epithelial cells (THLE3 and L02) were purchased from Cell Bank of Chinese Academy of Sciences (Shanghai, China). All HCC cells and L02 cells maintained in Dulbecco's modified Eagle's medium (DMEM; Life Technologies, Carlsbad, CA, USA) supplemented with 10% fetal bovine serum (FBS; Life Technologies). THLE3 cells were maintained in bronchial epithelial growth medium (Clonetics Corporation, Walkersville, MD), supplemented with 5 ng/ml epithelial growth factor, 70 ng/ml phosphoethanolamine and 10% fetal bovine serum.

### Patients and tumor tissues

A total of eight HCC fresh tissues and 80 human HCC tissues with matched adjacent normal tissues were obtained during surgery at Affiliated Hospital of Guiyang Medical College (Guiyang, China) between January 2004 and Jun 2014. Diagnosis was based on pathological evidence, and the specimens were immediately snap-frozen and stored in liquid nitrogen tanks. For the use of these clinical materials for research purposes, prior patients' consents and approval from the Institutional Research Ethics Committee were obtained.

### RNA extraction, reverse transcription, and real-time PCR

Total RNA from tissues or cells was extracted using TRIzol (Life Technologies) according to the manufacturer's instructions. Messenger RNA (mRNA) and miRNA were polyadenylated using a poly-A polymerase-based First-Strand Synthesis kit (TaKaRa Bio, DaLian, China ) and reverse transcription (RT) of total mRNA was performed using a PrimeScript RT Reagent kit (TaKaRa) according to the manufacturer's protocol. Complementary DNA (cDNA) was amplified and quantified on ABI 7500HT system (Applied Biosystems, Foster City, CA, USA) using SYBR Green I (Roche, Grenzach-Wyhlen, Germany). Supplementary Table 1 lists the primers used in the reactions. Primers for *U6* and miR-3127 were synthesized and purified by RiboBio (Guangzhou, China). *U6* or glyceraldehyde-3-phosphate dehydrogenase (*GAPDH*) was used as endogenous controls. Relative fold expressions were calculated with the comparative threshold cycle (2^−ΔΔCt^) method.

### Plasmid, small interfering RNA and transfection

The human miR-3127 gene was PCR-amplified from genomic DNA and cloned into a pMSCV-puro retroviral vector (Clontech, Tokyo, Japan). The reporter plasmid for quantitatively detecting the transcriptional activity of FOXO1 was ueing FHRE-Luc obtained from Addgene Inc. (Addgene plasmid 1789; Cambridge, MA, USA) as described by previous report [42]. The 3′-untranslated region (3′-UTR) region of the human PHLPP1, PHLPP2, INPP4A, and INPP5J was PCR-amplified from genomic DNA and cloned into pGL3 vectors (Promega, Madison, WI, US), and the plasmid phRL-tk was used as the internal control for transfection efficiency and cytotoxicity of test chemicals (Promega). A list of primers used in the reactions is presented in Supplementary Table 2. Antagomir-3127 and negative control RNA were synthesized and purified by RiboBio. Transfection of plasmids was performed using Lipofectamine 2000 (Life Technologies) according to the manufacturer's instructions.

### Tumor xenografts

All experimental procedures were approved by the Institutional Animal Care and Use Committee (IACUC) of Guiyang Medical College. The 6-week-old BALB/c-nu mice were randomly divided into three groups (n = 5 per group) and indicated cells (1 × 10^6^) were inoculated subcutaneously into the inguinal folds of the nude mice. In the experiment testing the anti-tumorigenicity effect of miR-3127, after 6 days for cells inoculation, animals were injected with 100 μl antagomir-3127 (1 mmol/L) or negative control through the lateral tail vein every three days for 4 weeks. Tumor volume was determined using an external caliper and calculated using the equation (L × W^2^)/2. The mice were sacrificed 35 days after inoculation and the tumors were excised and subjected to pathologic examination.

### 3-(4,5-Dimethyl-2-thiazolyl)-2,5-diphenyl-2H-tetrazolium bromide assay

Cells (2 × 10^3^) were seeded into 96 well plates and stained at the indicated time point with 100 μl sterile 3-(4,5-dimethythiazol-2-yl)-2,5-diphenyl tetrazolium bromide (MTT; Sigma-Aldrich, St Louis, MO) dye (at 0.5 mg/ml) for 4 h at 37°C, followed by removal of the culture medium and the addition of 150 μl dimethyl sulfoxide (Sigma-Aldrich). The absorbance was measured at 570 nm, with 655 nm used as the reference wavelength.

### Colony formation assay

Cells (0.2 × 10^3^) were plated into six well plates and cultured for 10 days. Colonies were then fixed for 5 min with 10% formaldehyde and stained with 1.0% crystal violet for 30 s.

### Anchorage-independent growth ability (soft agar) assay

Cells (3 × 10^3^) were suspended in 2 ml complete medium plus 0.3% agar (Sigma-Aldrich). The agar–cell mixture was plated as a top layer onto a bottom layer comprising 1% complete medium agar mixture. After 10 days culture, colony size was measured using an ocular micrometer and colonies >0.1 mm in diameter were counted.

### Flow cytometry analysis

Cells (5 × 10^5^) were harvested by trypsinization, washed in ice-cold phosphate-buffered saline and fixed in 80% ice-cold ethanol in phosphate-buffered saline (PBS). Before staining, cells were gently sedimented and resuspended in cold PBS. Bovine pancreatic ribonuclease (Sigma-Aldrich) was added to a final concentration of 2 μg/ml, and cells were incubated at 37°C for 30 min, followed by incubation with 20 μg/ml propidium iodide (Sigma-Aldrich) for 20 min at room temperature. Cell samples (2 × 10^4^) were then analyzed by Gallios flow cytometer (Beckman Coulter, Brea, CA, USA) and the data were analyzed using FlowJo 7.6 software (TreeStar Inc., Ashland, OR, USA).

### Western blotting analysis

Cell lysates were separated by 10% sodium dodecyl sulfate–polyacrylamide gel electrophoresis and transferred to polyvinylidene fluoride membranes (Millipore, Billerica, MA, USA). The membranes were probed with antibodies against cyclin D1, p21, p27, FOXO1, p-FOXO1 (S256), AKT, p-Akt (T308) and p-Akt (S473) (Cell Signaling Technology, Beverly, MA, USA), and GSK3β, p-GSK3β (S9), INPP4A and INPP5J (Abcam, Cambridge, MA, USA), and PHLPP1 and PHLPP2(Abnova, Taipei, Taiwan, China) overnight at 4°C, and then incubated with horseradish peroxidase–conjugated secondary antibodies (Cell Signaling Technology) for 1 h at room temperature. α-tubulin (Abcam) was used to correct for differences in protein loading from the control and experimental groups.

### Luciferase reporter assay

Cells were plated in 100-mm cell culture dishes, proliferating to 60–80% confluence after 24 h of culture. The reporter constructs were transfected using Lipofectamine 2000 (Life Technologies) according to the manufacturer's protocol. After 12-h incubation, the transfection medium was replaced; cells were harvested and washed with PBS, and lysed with passive lysis buffer (Promega). The cell lysates were analyzed immediately using a 96-well plate luminometer (Berthold Detection System, Pforzheim, Germany). Luciferase and Renilla luciferase were measured using a Dual-Luciferase Reporter Assay System (Promega) according to the manufacturer's instructions. The luciferase activity of each lysate was normalized to Renilla luciferase activity. The relative transcriptional activity was converted into fold induction above the vehicle control value.

### RNA immunoprecipitation (RIP)

Cells were cotransfected with a pIRESneo-FLAG/HA-Ago2 expression vector (Addgene plasmid 10822; Addgene Inc.) and miR-3127 mimic or non-targeting miRNA mimic (RiboBio). After 48-h transfection, cells were washed and lysed in radioimmunoprecipitation buffer (Sigma-Aldrich) containing 10% proteinase inhibitor cocktail (Sigma-Aldrich) and 1 mM phenylmethylsulfonyl fluoride (Sigma-Aldrich). A fraction of the whole cell lysate was used for RNA isolation, and the remaining lysate was subjected to immunoprecipitation (IP) using an antibody against Ago2 (Abcam) or immunoglobulin G (IgG) (Abcam). RNA from whole cell lysates and RNA IP (RIP) fractions was extracted with TRIzol (Life Technologies) according to the manufacturer's instructions. The relative levels of *PHLPP1, PHLPP2, INPP4A,* and *INPP5J* mRNA were determined using real-time RT-PCR as described above. The relative mRNA enrichment in the RIP fractions was computed based on the ratio of relative mRNA levels in the RIP fractions and the relative mRNA levels in the whole cell lysates (input).

### Akt activity assay

To measure kinase activities of in cells or tumor tissues, Akt was precipitated by a specific anti-Akt antibody. The immune-complexes were then incubated with a biotinylated peptide substrate, which became phosphorylated in the presence of activated Akt. The phosphorylated substrates, which reflected the activity of Akt kinase in the extract, was then quantified with the K-LISA Akt Activity Kit (Calbiochem, Darmstadt, Germany) that comprises a primary antibody recognizing the phosphorylated substrate peptides.

### Statistical analysis

All values are presented as means ± standard deviation (SD). Significant differences were determined using SPSS 16.0 software (SPSS, Chicago, IL, USA). Student's t-test was used to determine statistical differences. The chi-square test was used to analyze the relationship between miR-3127 expression and clinicopathological characteristics. Survival curves were plotted using the Kaplan Meier method and compared by log-rank test. *P* < 0.05 was considered significant.

## SUPPLEMENTARY MATERIAL, TABLES, FIGURES


