# Supplementary material for: MicroRNA-3127 promotes cell proliferation and tumorigenicity in hepatocellular carcinoma by disrupting of PI3K/AKT negative regulation
Source: Oncotarget. 2015 Jan 31;6(8):6359–72. doi: 10.18632/oncotarget.3438 (PMC4467442; doi:10.18632/oncotarget.3438)
Supplement: Supplementary file 1 [file oncotarget-06-6359-s001.pdf]

MicorRNA-3127 promotes cell proliferation and tumorigenicity in hepatocellular carcinoma by disrupting of PI3K/AKT negative regulation

Supplementary Materials and Methods

Supplementary Table 1: A list of primers used in the reactions for real-time RT-PCR.

**Real-time PCR primer:**	
PHLPP1-up	CCTGCCCGCTTATTTTGTAA
PHLPP1-dn	GGTTGTGTTGCACATCCAAG
PHLPP2-up	CTGCCAGACACCCTCTTCTC
PHLPP2-dn	AGCAGCTGCAGCATACTCAA
INPP4A -up	GCAGTGAGCTGCATACTCCA
INPP4A -dn	TGGTTCCCTCAATGATCTCC
INPP5J -up	CATTCTGCCTCAATCTTTTGC
INPP5J -dn	GACCCCAGTTGTCCACCC
cyclin D1 (CCND1)-up	GCCCTCGGTGTCCTACTTC
cyclin D1(CCND1)-dn	CTCCTCCTCGCACTTCTGTT
p21(CDKN1A)-up	AGGGGACAGCAGAGGAAGAC
p21(CDKN1A)-dn	GGCGTTTGGAGTGGTAGAAA
p27(CDKN1B)-up	GCAAAAATCCGAGGTGCTT
p27(CDKN1B)-dn	TTTACACAGCCCGAAGTGAA
GAPDH-up	GCACCGTCAAGGCTGAGAAC
GAPDH-dn	TGGTGAAGACGCCAGTGGA

Supplementary Table 2: A list of primers used in the reactions for clone PCR.

**Used for subcloning and plasmid construction:**	
pMSCV-miR-3127-up	GCCAGATCTATGACTAGGCACTATGGCTTATACC
pMSCV-miR-3127-dn	GCCGAATTCGCTTCTGACTGGTTGGATACAAG
pGL3- PHLPP1-up	GCCCCGCGGGCCGAGCTGTTTAACAAATAAACTA
pGL3- PHLPP1-dn	GCCCTGCAGTACTTGTAAATACTTGTAAATTTCATATTTATTG
pGL3-PHLPP2-up	GCCCCGCGGCTCTACGAGAATACAGCCTATCAAA
pGL3- PHLPP2-dn	GCCCTGCAGGGTTGAACATTAGACATCCATTAGAG
pGL3- INPP4A -up	GCCCCGCGGAAGGTTCAGGTTTCAGTATTCTCTC
pGL3- INPP4A -dn	GCCCTGCAGTCCAATGAAGTCACAAACATAGAAA
pGL3- INPP5J -up	GCCCCGCGGAGACGCCAACGAGAGAGGC
pGL3- INPP5J -dn	GCCCTGCAGTTTTCCTTGGTGCCTCCG

Supplementary Table 3: The relationship between miR-3127 and clinical pathological characteristics in 80 patients with HCC.

		miR-3127 expression		
Parameters	Number of cases	High	Low	*P* values
Sex				
Male	47	25	22	0.496
Female	33	15	18	
Age (years)				
<60	41	22	19	0.502
≥60	39	18	21	
Tumor size (cm)				
<5	22	6	16	0.012*
≥5	58	34	24	
Histologic grade				
High/moderate	33	12	21	0.041*
Poor	47	28	19	
Tumor status				
I - II	25	8	17	0.030*
III - IV	55	32	23	
Lymph node status				
Negative	34	13	21	0.070
Positive	46	27	19	
Microvascular invasion				
Negative	30	13	17	0.063
Positive	50	33	17	
Distant metastasis				
Negative	43	18	25	0.116
Positive	37	22	15	

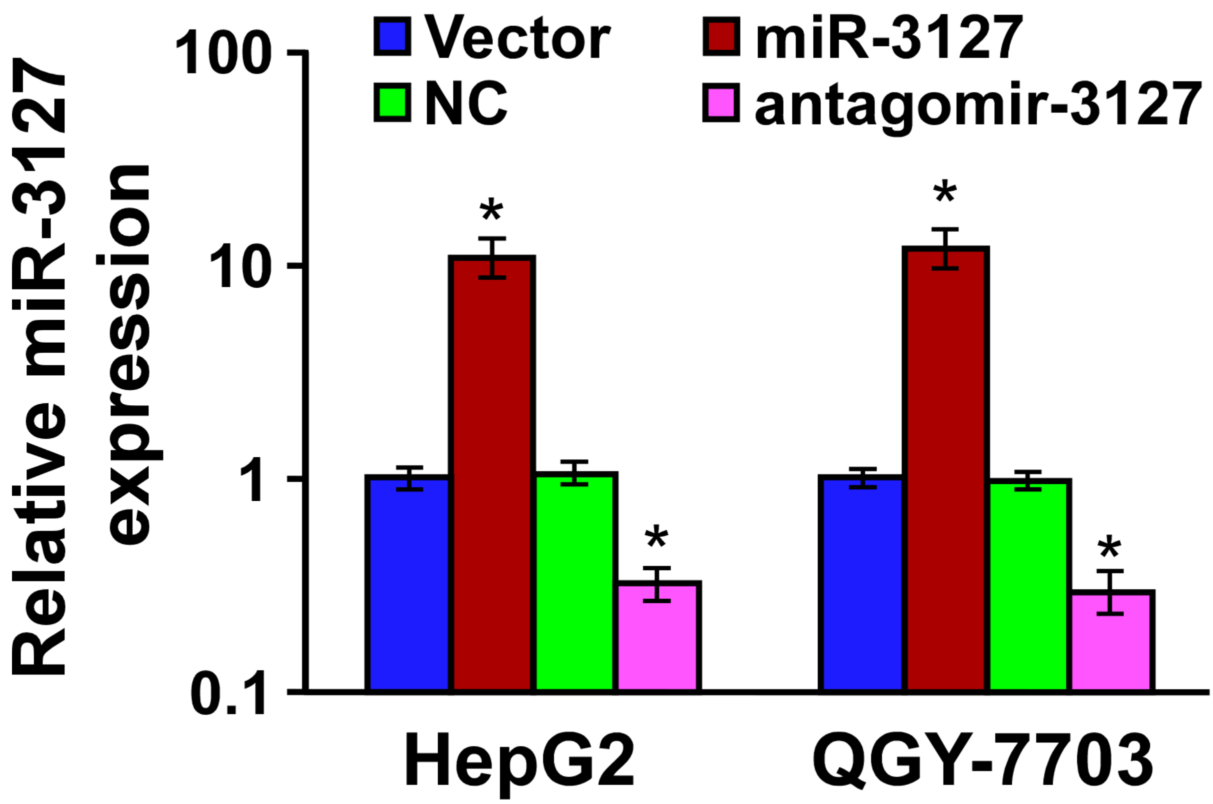

Supplementary Figure 1: Real-time PCR analysis of miR-3127 expression. MiR-3127 expression was examined in miR-3127–overexpressing and antagomir-3127 HepG2 and QGY-7703 cells and their respective control vectors. *U6* was used as the loading control. Bars represent the mean ± SD of three independent experiments. *$P < 0.05$. NC, negative control.

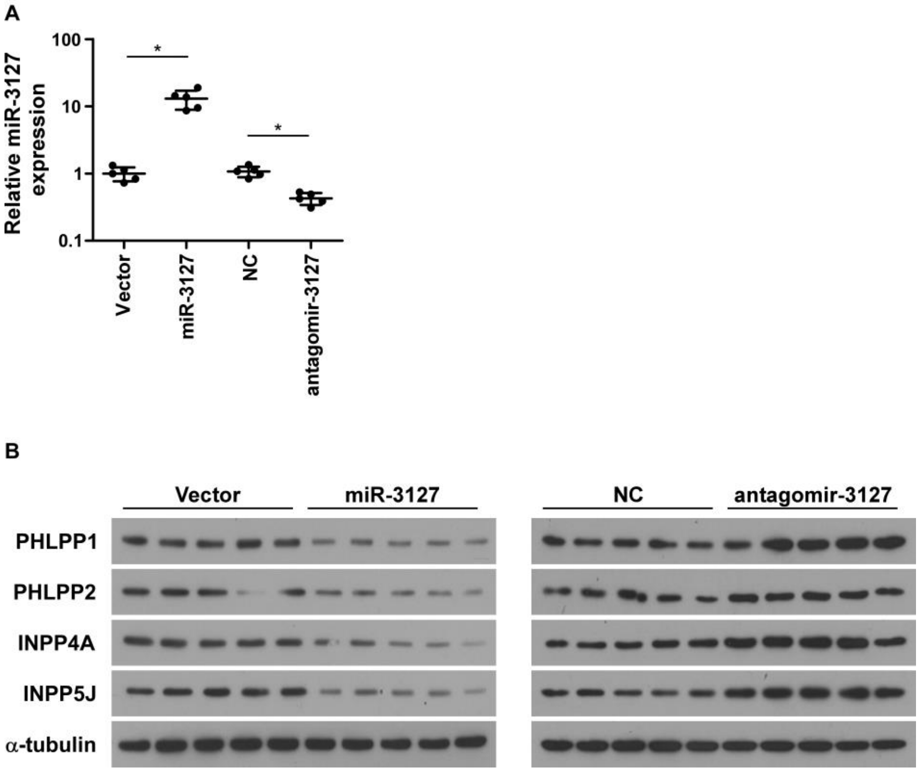

Supplementary Figure 2: A. Real-time PCR analysis of miR-3127 expression in xenografted tumor. B. Western blotting of PHLPP1, PHLPP2, INPP4A, and INPP5J expression in xenografted tumor. α-tubulin was used as the loading control. *P < 0.05.
